# Prevalence and Risk Factors of Giardia intestinalis Infestation and Assemblage of Isolates Among Monastery Primary School Children in Yangon, Myanmar

**DOI:** 10.7759/cureus.64155

**Published:** 2024-07-09

**Authors:** Yi Yi Myint, Win Pa Pa Aung, Maleewong Wanchai, Pewpan M. Intapan, Oranuch Sanpool, Aung Phyo Wai, Win Win Maw

**Affiliations:** 1 Microbiology, University of Medicine 2, Yangon, Yangon, MMR; 2 Parasitology, Khon Kaen University, Khon Kaen, THA; 3 Parasitology, Faculty of Medicine, Khon Kaen University, Khon Kaen, THA; 4 Parasitology, Faculty of Medicine, Khon Kaen University, Khon Khen, THA; 5 Mekong Health Science Research Institute, Faculty of Medicine, Khon Kaen University, Khon Khen, THA; 6 Microbiology, Faculty of Medicine, Shimane Univeristy, Izumo, JPN

**Keywords:** preschool children, myanmar, molecular identification, socioeconomic data, giardia intestinalis

## Abstract

Giardiasis* *is one of the major causes of diarrhea among children. To determine the prevalence, risk factors, and genotype of *Giardia intestinalis*, a cross-sectional descriptive study was done on stool samples of 462 children attending three monastery primary schools from North Okkalapa Township in Yangon, Myanmar from January 2016 to February 2019. Socioeconomic data were collected using a pre-tested questionnaire after obtaining informed consent. Direct wet mount, formalin-ether sedimentation, and trichrome staining techniques were used for the primary identification and then molecular identification was carried out by conventional polymerase chain reaction (PCR)-sequencing assay. *G. intestinalis *was identified in 11.7% (54/462) of students. There was no significant association with water source (p=0.948) and drinking untreated water (p=0.595). The infection was more common in children with low-educated parents, unsanitary garbage disposal practices, and no restrooms. All isolates were *G. intestinalis *assemblage B. This is the first study characterizing human isolates in a lower region of Myanmar, at the molecular level [MOU1]. These findings pointed out the high prevalence of *G. intestinalis *among primary school children from densely populated and low-resource settings.

## Introduction

Giardiasis, a major diarrheal disease found worldwide, is caused by flagellated protozoan *Giardia intestinalis* and occurs in 280 million people annually [1]. The prevalence of giardiasis varies from country to country and sometimes even within the same country, and the disease is more common among rural areas with potential sources of human infections such as pets, poultry, livestock, and other animals [2]. A large majority (35% to 70%) of infected individuals remain asymptomatic [3], but in some cases, infections can cause diarrhea, steatorrhea, nausea, vomiting, abdominal bloating, cramps, and malabsorption. Infection is also associated with malnutrition and physical and intellectual growth retardations (slow psychomotor and cognitive development) in school children in developing countries. Post-infectious irritable bowel syndrome (IBS), chronic fatigue syndrome, and arthritis can also occur [4-7].

Humans are the main reservoir of the parasite; asymptomatic persons may be the source of contamination as they can pass a large number of cysts in their stools [8]. Giardiasis is also a zoonotic infection; pet animals, agricultural animals, or wildlife animals are possible sources of zoonotic transmission, via ingestion of their excreta [8]. *Giardia* species are now considered the most common intestinal parasite in dogs and cats worldwide [9]. *Giardia* species easily spread through contaminated water and from person to person [10]. People who travel abroad to endemic areas are at a greater risk of exposure to *G. intestinalis* and children living in rural areas have a higher infection rate than those in urban areas [2, 11]. Males had a higher prevalence of *G. intestinalis* than females [11].

Infection with *Giardia* is common among Asian children including children in Southeast Asia especially in Cambodia, Malaysia, Myanmar, the Philippines, Laos, and Indonesia [2, 12]. In South Asia, India and Bangladesh have the most serious infections in children [12]. The pooled prevalence of *G. *i*ntestinalis* infection among Asian children was estimated at 15.1% [12]. From the 1980s onwards, *G. intestinalis* has been sub-classified into 8 assemblages (A-H) using Polymerase Chain Reaction (PCR) by detecting genes such as small subunit rDNA (SSU rDNA), variable surface protein (*vsp*), glutamate dehydrogenase (*gdh*), triose phosphate isomerase (*tpi*), elongation factor 1 alpha (ef1a), and beta guardian (b guardian) [13]. Assemblages A and B are common in humans; distribution varies in different parts of the world [13]. 

In Myanmar, there has been no study on the molecular characterization of *G. intestinalis, *and giardiasis is a common cause of morbidity in school-aged children. The current study aimed to determine the prevalence and the genetic characterization of *G. intestinalis* isolates from stool samples of the monastery primary school children in Yangon by microscopy and polymerase chain reaction (PCR) and its associated factors were also assessed. This study is the first report on the molecular identification of *Giardia* from Myanmar to comprehend more about the genetic diversity and transmission of giardiasis.

## Materials and methods

Study population and sampling procedure

The present study was carried out at the North Okkalapa Township, in the eastern part of Yangon, the most populous city of Myanmar. A multistage sampling method was used. Three monastery primary schools, each has at least 300 children were chosen again from selected townships by lottery method of randomization from 2016 to 2019. Written consent was obtained from the parents or guardians of the selected study children. A total of 462 individuals of both genders, with ages ranging from five years to 13 years, were investigated. A pre-tested questionnaire was used to identify known risk factors such as environmental, socio-demographic background, and behavioral factors. Parents or caregivers of children were asked to fill out the questionnaire and assist the children during sample collection.

Collection and storage of specimen

The single stool specimen was collected in a labeled clean dry mouth screw-capped bottle on the next day after getting the informed consent form. The stool specimen was transported at ambient temperature to the laboratory in the Department of Microbiology, University of Medicine 2. Samples that could not be processed within 24 hours were kept at 4˚C. Then, microscopic examination of stool samples was done by direct wet mount examination using NaCl 0.9% solution and Lugol’s iodine solution after concentration with formal ether sedimentation for detection of cyst and permanent staining with Trichrome to detect trophozoite and cyst.

Molecular identification of *G. intestinalis*

The fecal pellet was homogenized with disposable polypropylene pestles (Bellco Glass Inc., Vineland, NJ). These homogenized samples were subjected to DNA extraction using a QIAamp® DNA stool mini kit according to the manufacturer’s protocol. Two separate assemblage A-specific tpi-PCR and assemblage B- specific tpi-PCR were done [14]. Primers for assemblage A were F-tpiA 5'-GGAGACCG ACGAGCAAAGC-3' and R-tpiA 5'-CTTGCCAAGCGCCTCAA-3'. The PCR product is a fragment of approximately 148-bp tpi gene segments. For assemblage B, primers F-tpiB 5'-AATAGCAGC ACARAACGTGTATCTG-3’, and R-tpiB5'-CCCATGTCCAGCAGCATCT-3' were used and PCR yields a fragment of approximately 81-bp tpi gene segments. The PCR was carried out using a GeneAmp® PCR System 9700 (Applied Biosystems, Singapore). Both positive and negative controls were included in each PCR to validate the results. The sizes of the DNA amplicons were determined by electrophoresis on a 1.5% agarose gel (approximately 148-bp tpiAgene segments and approximately 81 bp assemblage B specific tpi ) [15].

Data Analysis

All data analysis was done by using Excel and Statistical Package for Social Science (SPSS) version 18.0 (SPSS Inc., Chicago, IL). The association between Giardia infection and environmental factors, children's behaviors, and clinical symptoms were analyzed using the chi-square test. A p-value of less than 0.05 was regarded as indicating statistical significance.

Ethical consideration

This research was done after obtaining ethical clearance from the Ethical and Research Committee of the University of Medicine 2, Yangon (16/ERC-3 (12-2016) on 9.12.2016. Written informed consent was obtained from the parents/guardians of participant children after a thorough explanation of the procedures of this study. Official approval was obtained from the Department of Education in the North Okkalapa Township for obtaining samples from school children.

## Results

Occurrence of *G. intestinalis* infection among the children studied

Among 462 children from three monastery primary schools, *G. intestinalis* was found in 54 children (11.7%). The detection rates were 11.5% (53/462) by direct microscopy, 11.7% (54/462) by formal-ether concentration method, and 11.3% (52/462) by trichome staining.

Molecular identification of *G. intestinalis* assemblage

All 54 (100%) microscopically proven *G. intestinalis* positive samples were successfully amplified with assemblage B specific *tpi* primers but not with assemblage A specific *tpi* primer set.

Background characteristics and behavior of the children, parents’ status, and environmental factors

The study group comprised of 271 (58.7%) male and 191 (41.3%) female schoolchildren (see Table 1). We grouped into 2 categories according to age; 319 (69%) were in the 10 years and under age group and 143 (31%) were in the over 10 years age group. Among 462 children, 397 (85.9%) attended the school from home, and only 65 (14.1%) children stayed at the monastery (Table 1).

**Table 1:** Distribution of G. intestinalis infections in relation to background characteristics of the children, and parents’ education status (n=462)

Variable	Category	*Giardia* Infection		χ^2^	P value
		Positive	Negative		
Age group	≤10 years	39(12.2%)	280(87.8%)	0.288	0.591
	>10 years	15(10.5%)	128(89.5%)		
Sex	Male	33(12.2%)	238(87.8%)	0.152	0.697
	Female	21(11%)	170(89%)		
Grade	Grade1	4(7.7%)	48(92.3%)	8.561	0.073
	Grade 2	7(8.4%)	76(91.6%)		
	Grade 3	15(20.8%)	57(79.2%)		
	Grade 4	15(13.2%)	99(86.8%)		
	Grade 5	13(9.2%)	128(90.8%)		
Monastery	DMMDN	23(14.3%)	138(85.7%)	1.62	0.445
	DMDY	14(10.4%)	120(89.6%)		
	MK	17(10.2%)	150(89.8%)		
Residence	Home	47(11.8%)	350(88.2%)	0.062	0.804
	Monastery	7(10.8%)	58(89.2%)		
Mother’s education status	Up to Primary school	37 (13%)	247 (87%)	7.562	0.182
	Middle school	12 (13.8%)	75 (86.2%)		
	High school	4 (5.2%)	73 (94.8%)		
	University/Graduate	0 (0%)	10 (100%)		
Father’s education status	Up to Primary school	20 (10.5%)	170 (89.5%)	4.75	0.446
	Middle school	17 (16.5%)	86 (83.5%)		
	High school	12 (12.0%)	88 (88.0%)		
	University/Graduate	0 (0%)	16 (100%)		

With regard to the education level, more than half of the mothers were primary school leavers (62.0%; 284/458). Only 19% (87/458) and 16.8% (77/458) of the mothers had middle and high school levels of education, respectively. As for the fathers, primary, middle, and high school leavers were 46.5% (190/409), 25.2% (103/409), and 24.4% (100/409), respectively. Only a few numbers of parents obtained an academic degree (2.2%; 10/458 of mothers and 3.9%; 16/409 of fathers).

The majority of the parents were casual laborers (52%; 238/458 of mothers and 77.7%; 318/409 of fathers). The dependent rate (without a job) is higher in mothers (40.4%; 185/458 vs 4.9%; 20/409 of fathers). Only 5.5% (25/458) of mothers and 12.7% (52/409) of fathers were self-employed or staff of private companies. A small portion, 2.2% (10/458) of mothers and 4.6% (19/409) of fathers were government staff.

More than half of study children used untreated water for drinking and 33.5% (155/462) practiced unsanitary disposal like dumping or throwing into the river. Among 462 study children, the minority had no restrooms and never practiced hand washing after the toilet (3.9% (18/462) each). Similarly, 7.4% (34/462) of the children never practiced hand washing before meals (Table 2). According to the association factors evaluation, Children's education level and proper waste disposal are positively correlated with the infection rate (p-value = 0.073 and 0.035).

**Table 2:** Distribution of G. intestinalis infections in relation to environmental factors and behavior of the children

Variable		Category	*Giardia* Infection		χ^2^	P value
			Positive	Negative		
Environmental factors						
Domestic animal		Present	17(9.8%)	154 (90.2%)	0.929	0.335
		Absent	37 (12.8%)	252 (87.2%)		
Water supply		Public	38 (11.6%)	290 (88.4%)	0.364	0.948
		Tube well	10 (13.2%)	66 (86.8%)		
		Shallow well	2 (8.7%)	21 (91.3%)		
		Lake	4 (11.4%)	31 (88.6%)		
Drinking water		Purified	20 (13.2%)	132 (86.8%)	1.891	0.595
		Boiled	6 (16.7%)	30 (83.3%)		
		Filter	27 (10.4%)	233 (89.6%)		
		Raw	1 (7.1%)	13 (92.9%)		
Excreta		Latrine with flush	20 (11.5%)	154 (88.5%)	0.45	0.799
		Pit latrine	31 (11.5%)	239 (88.5%)		
		No latrine	3 (16.7%)	15 (83.3%)		
Garbage		Sanitary	29 (9.6%)	278 (90.4%)	4.456	0.035
		Unsanitary	25 (16.1%)	130 (83.9%)		
Behavioral factors						
Hand washing after toilet		Always	39 (11.8%)	291 (88.2%)	0.587	0.746
		Occasional	12 (10.5%)	102 (89.5%)		
		Never	3 (16.7%)	15 (83.3%)		
Hand washing before meal		Always	38 (12.1%)	277 (87.9%)	1.207	0.547
		Occasional	14 (12.4%)	99 (87.6%)		
		Never	2 (5.9%)	32 (94.1%)		
Nail biting habit		Present	5 (7.2%)	64 (92.8%)	1.551	0.213
		Absent	49 (12.5%)	344 (87.5%)		
Eating raw vegetables		Present	37 (9.6%)	253 (87.2%)	0.864	0.352
		Absent	17 (16.1%)	155 (90.1%)		

Distribution of *Giardia intestinalis* infection in relation to clinical symptoms

Among the 54 *G. intestinalis*-positive children, 17(31.5%) were symptomatic and 37 (68.5%) were asymptomatic. Out of 408 *G. intestinalis*-negative children, 110 (27%) children were found to have symptoms. This was statistically not significant (P = 0.484). Moreover, among the 33 children who were infected only with *G. intestinalis* (without other intestinal parasitic infections), 10 (30.3%) were symptomatic and more than half (23/33; 69.7%) were asymptomatic.

## Discussion

The prevalence of *G. intestinalis *in Yangon, Myanmar children was less than 10% (2010-2016) [16, 17]. The prevalence of G. intestinalis infection in this study (54/462, 11.7%) was higher than that of other previous studies in Myanmar where the prevalence was 4.3% among school children in South Dagon and HlaingTharYar districts, Yangon, in 2016, and 8% in primary school children in Kanpya village, Magway division, in 2010 [16, 17]. The prevalence of *G. intestinalis* in Myanmar varies depending on geographical area, socioeconomic status, source of water supply, differences in age of the target population, and adopted detection methods.

The overall prevalence of *Giardia* infections observed in this study is comparable with studies conducted in neighboring countries such as the Cambodian study (11.2%) and rural Malaysia study (11.6%) [18, 19]. On the contrary, the Giardia prevalence in our study is higher than the prevalence reported in China (0.72%) [20] and Thailand (3.5%) [21]. This is the first study characterizing human isolates of *G. intestinalis* in Myanmar, at the molecular level. Two separate assemblages specific *tpi* PCR showed isolates of *G. intestinalis* assemblage B type. The predominance of assemblage B was also found in Asia such as Malaysia [22], Bangladesh [23], India [24], Thailand [2], and China [25]. The distribution of *G. intestinalis* assemblages varied throughout different geographical places; however, Myanmar's genotype is comparable to its neighboring countries.

This study was done on primary monastery school children with the age ranges of five to 13 years. The majority of the parents in the present study are casual laborers and daily wage earners working in vegetable plantations, unskilled laborers in factories or construction sites and trishaw or cycle carry drivers and selling goods or raw vegetables in wet markets without any stable income. Moreover, less than 5% of the parents were university graduates, and their children showed a lower *G. intestinalis* infection rate, although statistically not significant. As the participants in this study are from low-income communities (slums), there is a lack of basic facilities, and inadequate sanitation, and hygiene facilities. More than half of the children studied drink untreated water, and a few houses are still using shallow wells and lakes as the water source.

The parasite is associated with poverty, poor sanitation, lack of clean and safe drinking water supply, and poor personal hygiene [12]. As giardiasis is a major waterborne infection, the source of water supply and drinking water quality is important [12]. However, the present study revealed that there was no significant association between *G. intestinalis* infection and domestic water sources and drinking untreated water, and with behavior of the study children such as hand washing before meals and after toilet, nail-biting habits, and consuming raw vegetables. On the contrary, although statistically not significant, G. intestinalis infection was more common in children with no latrines (3, 16.7%) and children with unsanitary garbage disposal practices.

Different from our findings, not washing hands before eating, not washing hands after playing with animals, not boiling water before consumption, bathing in the river, and not wearing shoes when outside are the significant risk factors for *G. intestinalis* infection in Malaysia [19]. In this study, the majority of children with *G. intestinalis* infections were asymptomatic. This finding is in agreement with previous studies where a large majority of individuals (35% to 70%) infected with giardiasis remain asymptomatic [3, 26]. The limitation of this study is that only one stool sample was taken from the study population for detection of Giardia cyst although it is recommended to take three times to increase sensitivity [27]. Moreover, it was suggested that the research population and sampling area should be increased to include additional districts of Myanmar. This study highlights the monitoring of giardiasis in Myanmar and suggests more research on the genotype level and their potential for zoonotic transmission.

## Conclusions

This study revealed that the prevalence of *G. intestinalis* infections is high among school children in Yangon, Myanmar where *G. intestinalis* was not considered as an important pathogen and neglected, even in settings where mass treatment is underway against intestinal helminthic infections.
